# Supplementary figures and images for: Ancient DNA of the Don-Hares Assumes the Existence of Two Distinct Mitochondrial Clades in Northeast Asia
Source: Genes (Basel). 2023 Mar 12;14(3):700. doi: 10.3390/genes14030700 (PMC10047931; doi:10.3390/genes14030700)

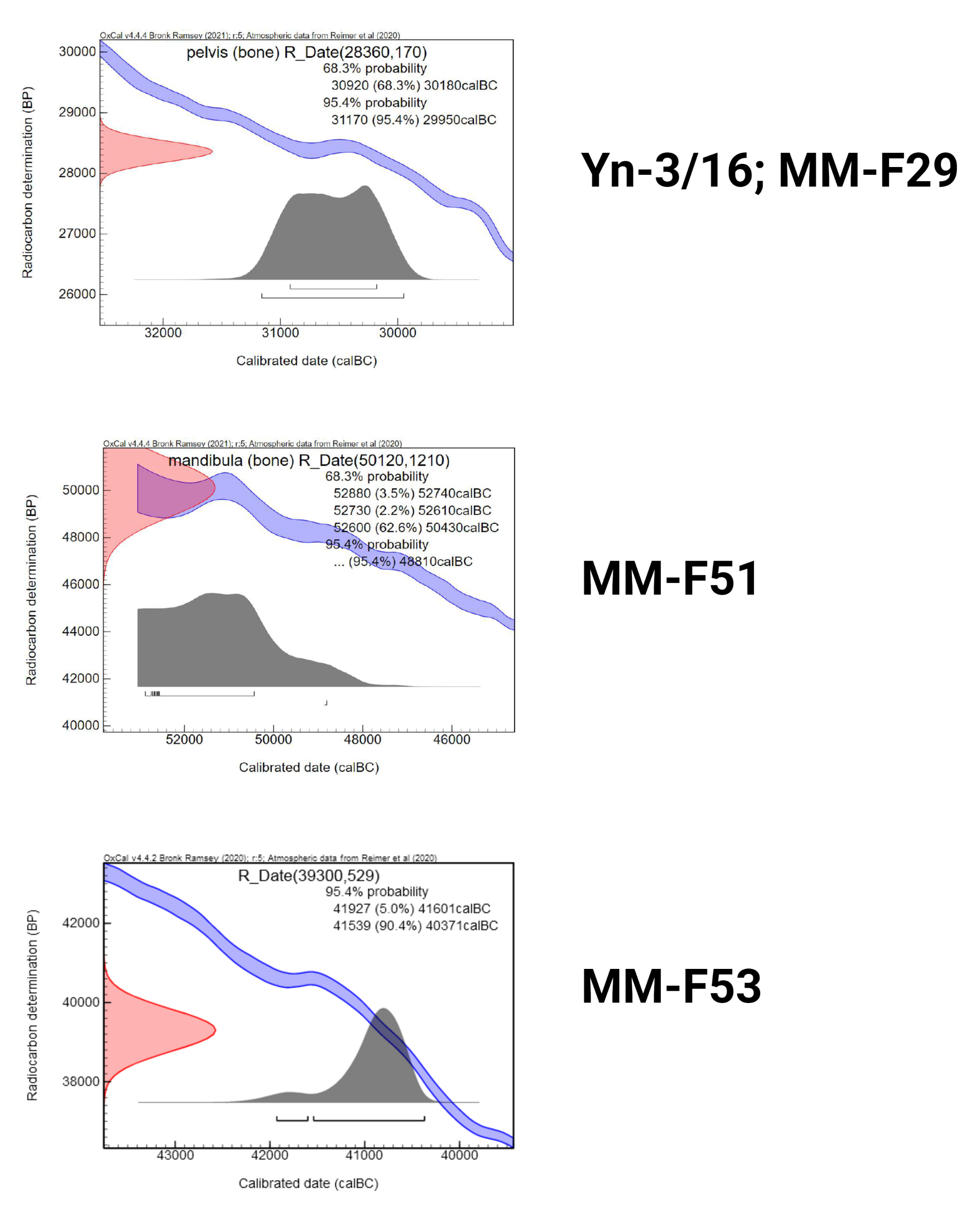

Supplement: Supplementary file 1 [file genes-14-00700-s001.zip › supplementary_files/Figure_S1.png]
